# Is There Any Association between PEEP and Upper Extremity DVT?

**DOI:** 10.1155/2015/614598

**Published:** 2015-04-02

**Authors:** Farah Al-Saffar, Ena Gupta, Furqan Siddiqi, Muhammad Faisal, Lisa M. Jones, Vandana Seeram, Mariam Louis, James D. Cury, Abubakr A. Bajwa, Adil Shujaat

**Affiliations:** ^1^Department of Medicine, University of Florida College of Medicine, Jacksonville, FL 32209, USA; ^2^Division of Pulmonary, Critical Care and Sleep Medicine, University of Florida College of Medicine, Jacksonville, FL 32209, USA

## Abstract

*Background*. We hypothesized that positive end-exploratory pressure (PEEP) may promote venous stasis in the upper extremities and predispose to upper extremity deep vein thrombosis (UEDVT). *Methods*. We performed a retrospective case control study of medical intensive care unit patients who required mechanical ventilation (MV) for >72 hours and underwent duplex ultrasound of their upper veins for suspected DVT between January 2011 and December 2013. *Results*. UEDVT was found in 32 (28.5%) of 112 patients. Nineteen (67.8%) had a central venous catheter on the same side. The mean ± SD duration of MV was 13.2 ± 9.5 days. Average PEEP was 7.13 ± 2.97 cm H_2_O. Average PEEP was ≥10 cm H_2_O in 23 (20.5%) patients. Congestive heart failure (CHF) significantly increased the odds of UEDVT (OR 4.53, 95% CI 1.13–18.11; *P* = 0.03) whereas longer duration of MV (≥13 vs. <13 days) significantly reduced it (OR 0.29, 95% CI 0.11–0.8; *P* = 0.02). Morbid obesity showed a trend towards significance (OR 3.82, 95% CI 0.95–15.4; *P* = 0.06). Neither PEEP nor any of the other analyzed predictors was associated with UEDVT. *Conclusions*. There is no association between PEEP and UEDVT. CHF may predispose to UEDVT whereas the risk of UEDVT declines with longer duration of MV.

## 1. Introduction

Deep vein thrombosis (DVT) is not uncommon in critically ill patients, is often overlooked in such patients, and may develop despite prophylaxis [1]. It may develop in the lower extremity or, less commonly, in the upper extremity. Recently, it has become recognized that upper extremity DVT (UEDVT) in critically ill patients is also frequently complicated by pulmonary embolism (PE) albeit not as often as lower extremity DVT. In a recent study, as many as 15% of critically ill patients with non-leg DVT, the majority (94.5%) of which were UEDVT, developed PE [2]. Moreover, UEDVT may be associated with central venous catheter- (CVC-) related sepsis. In one study, the risk of catheter-related sepsis was 2.6-fold higher when UEDVT occurred [3]. Intensive care unit (ICU) and hospital length of stay are significantly prolonged in patients who develop UEDVT [2]. There is a need to recognize risk factors for UEDVT, so such DVT may be diagnosed and treated early and the complications of PE and catheter-related sepsis avoided.

Few studies have reported on risk factors for UEDVT in critically ill patients. Malignancy, central venous catheterization, and obesity were found to be significant risk factors for UEDVT in such patients [1, 4, 5]. Mechanical ventilation may also be a risk factor [6]. Paralysis, sedation, and immobility associated with mechanical ventilation may play a role in the development of DVT by promoting venous stasis [6]. Moreover, positive end-exploratory pressure (PEEP), particularly high levels, may promote venous stasis in the upper extremities by impeding venous return to the chest. Central venous pressure (CVP) increases along with increases in intrathoracic pressure, for example, with positive pressure ventilation or hyperinflation during mechanical ventilation. As a result, the pressure gradient for systemic venous return decreases, decelerating venous blood flow [7, 8]. Central venous pressure increases with PEEP in mechanically ventilated patients [8]. We, therefore, hypothesized that PEEP may be a risk factor for UEDVT.

## 2. Material and Methods

We performed a retrospective case control study of medical ICU patients who required mechanical ventilation for >72 hours and underwent duplex ultrasound exams of their upper veins for suspected DVT between January 2011 and December 2013. The study (UFJ 2014-025) was approved by the Institutional Review Board Committee at the University of Florida in Jacksonville.

The diagnosis of DVT was based on the visualization of an intravascular thrombus, incompressibility of the vein by probe pressure, absence of spontaneous flow by Doppler, and absence of variation in flow with respiration. The diagnosis of UEDVT required direct visualization of the thrombus and one or more of the other signs in the internal jugular, subclavian, axillary, brachiocephalic, or brachial veins. The central venous catheters (CVC) used at our institution are made of oligon material and heparin-coated. Catheter-related UEDVT was defined as DVT in an upper extremity vein in which CVC or peripherally inserted catheter (PICC) was in place at the time of diagnosis or within the preceding 72 hours.

We collected the following data: demographics, any known risk factors for venous thromboembolism (VTE), any use of prophylactic or therapeutic anticoagulation, duration of mechanical ventilation, and PEEP. We looked for the following known risk factors for DVT: malignancy, prior history of VTE, congestive heart failure (CHF), chronic obstructive pulmonary disease, other lung disease, atrial fibrillation, end-stage renal disease, sepsis, CVC or PICC, and morbid obesity. We looked for the possibility of an association between PEEP and UEDVT by examining the average PEEP over a period of 3 days and 7 days prior to venous duplex US exam and by comparing average PEEP <10 cm H_2_O versus ≥10 cm H_2_O over a period of 3 days and 7 days prior to the exam. “Average” PEEP was obtained by multiplying the median PEEP over a 24-hour period by the number of days, that is, 3 and 7, and dividing the product by the number of days. We used STATA 13 to summarize and analyze the data. Associations were expressed in terms of odds ratios (OR) and 95% confidence interval (95% CI). Fischer's exact test was used to estimate *P* values where cell counts were ≤5. The alpha level of significance was set to be 0.05.

## 3. Results

There was a total of 112 patients who required mechanical ventilation for >72 hours and underwent venous duplex exams for suspected UEDVT. The mean age was 57.6 ± 17.6 years and 51% of them were male. The characteristics of the study population are listed in Table 1. The majority of them had a CVC or a PICC (*n* = 89, 80%) and sepsis (*n* = 68, 61%) and were receiving pharmacologic prophylaxis (*n* = 83, 74%) with low molecular weight heparin or unfractionated heparin 5000 units subcutaneously every 8 hours. Average PEEP ≥10 cm H_2_O was used in only a few (*n* = 23, 20.5%) patients. Upper extremity DVT was found in 32 (28.5%) patients. Twenty-eight (87.5%) of the patients with UEDVT had a CVC or a PICC. Of those, 19 (67.8%) were on the same side as the UEDVT.

Univariable analysis failed to reveal any association between PEEP and UEDVT. Malignancy was more common in patients with UEDVT compared to those without it (18.8% versus 12.8%) and so were sepsis (68.7% versus 57%) and central venous catheterization (87.5 versus 76.3%). However, none of these were significantly associated with UEDVT (Table 2).

Morbid obesity was more common in patients with UEDVT compared to those without it (19.3% versus 6.3%) and was significantly associated with UEDVT (OR 3.56, 95% CI 1–12.65; *P* = 0.04). Similarly, CHF was more common in patients with UEDVT (21.8% versus 6.3%) and was also significantly associated with UEDVT (OR 4.2, 95% CI 1.22–14.42; *P* = 0.023). A shorter duration of mechanical ventilation was found in patients with UEDVT (10.32 ± 6.51 versus 14.3 ± 10.24 days). CHF and shorter duration of mechanical ventilation remained significant predictors of UEDVT after ORs were adjusted for PEEP (being the primary outcome variable of interest) as well as sepsis (Table 3).

## 4. Discussion

Upper extremity DVT was found in 32 (28.5%) of 112 patients who required mechanical ventilation for >72 hours and underwent venous duplex exams for suspected UEDVT. We did not find any association between PEEP and UEDVT. We examined PEEP over 3 days and 7 days prior to venous duplex US exams and high versus low PEEP but neither was found to be a significant predictor of UEDVT. There are a few possible explanations. The level of PEEP may not have been high enough even though PEEP ≤10 cm H_2_O may increase CVP [8]. There is not a large reservoir of blood in the upper extremities that is at risk for venous stasis unlike the lower extremities. The veins in the upper extremities are closer to the chest and may not be prone to venous stasis despite high levels of PEEP. The majority of catheters in our study were heparin-coated central venous catheters.

Of the other potential risk factors for DVT that were analyzed, morbid obesity, CHF, and duration of mechanical ventilation were found to be significant. Congestive heart failure was a significant predictor of UEDVT despite the small number of patients in our study suggesting it is a strong risk factor. Congestive heart failure is a known risk factor for lower extremity DVT [9]; however, no previous study has reported it as a risk factor for UEDVT. Interestingly, the risk of UEDVT declined with longer duration of mechanical ventilation. This finding is supported by another study in which the majority of UEDVT was found in the first 2 weeks of mechanical ventilation [1]. It is possible that higher levels of sedation and paralysis used in the first few days of mechanical ventilation lead to immobility and predispose to venous stasis. Moreover, if heart failure predisposes to UEDVT, then it is plausible that as heart function improves with mechanical ventilation, the risk of UEDVT should decline. In fact, the duration of mechanical ventilation became more significant when adjusted for CHF.

Similar to a recent study [5], obesity was found to be a significant risk factor for UEDVT. Of note, BMI ≥ 35 was found to be significant in that study whereas BMI > 40 was significant in ours. We used a cut-off of 40 because it defines morbid obesity. However, there was only a trend towards significance when morbid obesity was adjusted for sepsis and PEEP. Contrary to other studies, malignancy and central venous catheterization were not found to be significant risk factors for UEDVT. Malignancy was not found to be significant despite a prevalence (13.5%) similar to another study in which this risk factor was significant [1]. This is possibly because in our study ultrasonography was done when UEDVT was clinically suspected whereas in the other study it was done every 7 days to screen for UEDVT [1]. Central venous catheterization was not found to be a significant risk factor for UEDVT possibly because the majority (79%) of our patients had a CVC or PICC and the CVC were heparin-coated. Moreover, since ultrasonography was only done when UEDVT was clinically suspected, cases of asymptomatic UEDVT were missed. Furthermore, since we defined central venous catheter as a risk factor for UEDVT if one had been in place within 3 days prior to diagnosis of UEDVT and catheter-related UEDVT may resolve with removal of the catheter alone, it is possible that some cases of UEDVT were missed. Lastly, it is possible that the duration of central venous catheterization is more important than the presence of a catheter* per se* [1]. We did not examine the duration because CVC are usually removed or replaced by day 7 in our medical ICU.

It is worth mentioning that the 28.5% rate of UEDVT in our study was higher than usual [5] because thromboprophylaxis was used in only 74% of our patients. More importantly, thromboprophylaxis was not predictive of UEDVT. This is not surprising because UEDVT may develop in as many as 11% of critically ill patients despite such prophylaxis [5].

Our study is not without limitations. It is retrospective and does not exclude the possibility of prevalent UEDVT at the time of admission to the medical ICU since patients were not screened. However, it is unlikely this would have affected the results since only 0.2% of non-leg DVT was prevalent DVT in the PROTECT study [2]. Our study did not look at outcomes like PE, catheter-related sepsis, length of stay, and mortality. However, it was not designed to examine outcomes. Lastly, our study failed to prove our hypothesis. Whether PEEP has any association with UEDVT remains to be proven. Perhaps, it may be worthwhile to test the hypothesis in the patient population in whom higher levels of PEEP may be used, that is, patients with acute respiratory distress syndrome.

## 5. Conclusions

Nevertheless, our study has some strengths. It shows that UEDVT is quite common in the medical ICU patients who require mechanical ventilation for >72 hours, being found in as many as 29% of patients in whom it is suspected. Moreover, it shows that CHF is a risk factor for UEDVT and suggests that morbid obesity may be another one. We believe that recognition of these two risk factors is important for two reasons. First, presence of one or both in medical ICU patients who require mechanical ventilation for >72 hours should prompt physicians to consider diagnosis of UEDVT earlier and allow them to treat it earlier to possibly prevent the complications of PE and catheter-related sepsis in such patients. Second, the development of UEDVT despite the use of pharmacologic prophylaxis in the majority of the patients in our study suggests that perhaps the subcutaneous route is unreliable in the edematous CHF patients and that perhaps higher doses of subcutaneous heparin may be required in the morbidly obese patients.

## Figures and Tables

**Table 1:** Characteristics of the study population.

Predictors	Mean ± SD or *N* (%)
Age (years)	57.58 ± 17.58
Male gender	57 (50.9)
BMI ≥ 40 (kg/m^2^)	11 (11.6)
Atrial fibrillation	9 (8)
Bedridden prior to admission	12 (10.7)
CAD	10 (8.9)
CHF	12 (10.7)
COPD	19 (17)
CVC/PICC	89 (79.5)
DM	44 (39.3)
ESRD	13 (11.6)
Home anticoagulation	34 (30.4)
INR on admission	1.77 ± 2.25
Malignancy	15 (13.5)
Other lung disease	11 (9.8)
Sepsis	68 (60.7)
VTE in the past	8 (7.2)
VTE thromboprophylaxis in hospital	83 (74.1)
Duration of MV (days)	13.20 ± 9.49
Average PEEP over 7 days (cm H_2_O)	7.13 ± 2.97
Average PEEP over 3 days (cm H_2_O)	7.10 ± 3.08
Average PEEP over 7 days ≥10 cm H_2_O	21 (18.8)
Average PEEP over 3 days ≥10 cm H_2_O	23 (20.5)

BMI: body mass index, CAD: coronary artery disease, CHF: heart failure, COPD: chronic obstructive airway disease, CVC: central venous catheter, DM: diabetes mellitus, ESRD: end-stage renal disease, MV: mechanical ventilation, *N*: number, PEEP: positive end expiratory pressure, PICC: peripherally inserted central catheter, SD: standard deviation, and VTE: venous thromboembolism.

**Table 2:** Univariable analysis of predictors of upper extremity deep vein thrombosis.

Predictors	Odds ratio	95% confidence interval	*P* value
Age (years)	1.023	0.998–1.049	0.065
Male gender	0.798	0.351–1.815	0.59
BMI ≥ 40 (kg/m^2^)	3.552	1–12.654	**0.04**
Atrial fibrillation	1.276	0.299–5.444	0.71^*^
Bedridden prior to admission	1.878	0.564–6.604	0.29
CAD	1.079	0.260–4.459	1^*^
CHF	4.2	1.223–14.422	**0.023**
COPD	0.619	0.189–2.032	0.58^*^
CVC/PICC	2.180	0.678 – 7.006	0.21
DM	0.615	0.257–1.466	0.27
ESRD	2.406	0.740–7.822	0.14
Home anticoagulation	1.384	0.569–3.364	0.47
INR on admission	0.996	0.827–1.198	0.94
Malignancy	1.794	0.581–5.538	0.31
Other lung disease	0.526	0.107–2.580	0.42^*^
Sepsis	1.626	0.682–3.878	0.266
VTE in the past	0.822	0.157–4.305	1^*^
VTE thromboprophylaxis in hospital	0.852	0.338–2.141	0.73
Duration of MV (days)	0.944	0.044–0.893	**0.05**
Duration of MV ≥ 13 days	0.339	0.131–0.876	**0.026**
Average PEEP over 7 days (cm H_2_O)	0.988	0.859–1.136	0.86
Average PEEP over 3 days (cm H_2_O)	0.964	0.839–1.108	0.60
Average PEEP over 7 days <10 cm H_2_O	1	0.349–2.859	1
Average PEEP over 3 days ≥10 cm H_2_O	0.459	0.143–1.474	0.21

BMI: body mass index, CAD: coronary artery disease, CHF: congestive heart failure, COPD: chronic obstructive airway disease, CVC: central venous catheter, DM: diabetes mellitus, ESRD: end-stage renal disease, PEEP: positive end expiratory pressure, PICC: peripherally inserted central catheter, and VTE: venous thromboembolism.

^*^ *P* value was calculated with Fischer's exact test.

**Table 3:** Odds ratios for significant predictors of upper extremity deep vein thrombosis adjusted for sepsis and PEEP.

Predictors	Adjusted odds ratio (95% confidence interval)	*P* value
BMI ≥ 40 (kg/m^2^)	3.818 (0.947–15.401)	0.060
CHF	4.321 (1.018–18.337)	**0.047**
Sepsis	1.525 (0.599–3.881)	0.376
Duration of MV ≥ 13 days	0.292 (0.105–0.816)	**0.019**
Average PEEP over 7 days (cm H_2_O)	1.067 (0.916–1.243)	0.408

BMI: body mass index, CHF: congestive heart failure, MV: mechanical ventilation, and PEEP: positive end-expiratory pressure.

## References

[1] Ibrahim E. H., Iregui M., Prentice D., Sherman G., Kollef M. H., Shannon W. (2002). Deep vein thrombosis during prolonged mechanical ventilation despite prophylaxis. *Critical Care Medicine*.

[2] Lamontagne F., McIntyre L., Dodek P. (2014). Nonleg venous thrombosis in critically ill adults: a nested prospective cohort study. *JAMA Internal Medicine*.

[3] Timsit J. F., Farkas J. C., Boyer J. M. (1998). Central vein catheter-related thrombosis in intensive care patients: Incidence, risks factors, and relationship with catheter-related sepsis. *Chest*.

[4] Hirsch D. R., Ingenito E. P., Goldhaber S. Z. (1995). Prevalence of deep venous thrombosis among patients in medical intensive care. *The Journal of the American Medical Association*.

[5] Blaivas M., Stefanidis K., Nanas S. (2012). Sonographic and clinical features of upper extremity deep venous thrombosis in critical care patients. *Critical Care Research and Practice*.

[6] Cook D., Attia J., Weaver B., McDonald E., Meade M., Crowther M. (2000). Venous thromboembolic disease: an observational study in medical-surgical intensive care unit patients. *Journal of Critical Care*.

[7] Pinsky M. R., Peacock A. J., Naeije R., Rubin L. J. (2011). Hemodynamic effects of changes in intrathoracic pressure. *Pulmonary Circulation*.

[8] Cao F., Liu X.-F., Chen R.-L., Wang X.-C. (2008). Effect of positive end-expiratory pressure on central venous pressure and common iliac venous pressure in mechanically ventilated patients. *Chinese Critical Care Medicine*.

[9] Beemath A., Stein P. D., Skaf E., Al Sibae M. R., Alesh I. (2006). Risk of venous thromboembolism in patients hospitalized with heart failure. *The American Journal of Cardiology*.

